# Characterization and Therapeutic Potential of a Novel Lytic Bacteriophage vB_Kp_H122 Targeting Multidrug‐Resistant Hypervirulent *Klebsiella pneumoniae* of K57 Capsular Type

**DOI:** 10.1155/tbed/8429457

**Published:** 2026-08-13

**Authors:** Cuilong Fan, Qiu Xu, Stefan Schwarz, Yaowei Liu, Susu Du, Xiaofeng Lu, Yuxuan Yuan, Longhua Lin, Shuangyu Xie, Fang Xie, Wanjiang Zhang

**Affiliations:** ^1^ State Key Laboratory of Animal Disease Control and Prevention, Harbin Veterinary Research Institute, Chinese Academy of Agricultural Sciences, Harbin, China, caas.cn; ^2^ Institute of Microbiology and Epizootics, Centre for Infection Medicine, School of Veterinary Medicine, Freie Universität Berlin, Berlin 14163, Germany, fu-berlin.de; ^3^ Veterinary Centre for Resistance Research (TZR), School of Veterinary Medicine, Freie Universität Berlin, Berlin 14163, Germany, fu-berlin.de

**Keywords:** antibiofilm activity, bacteriophage, biological characteristics, genome analysis, *Klebsiella pneumoniae*, phage therapy

## Abstract

*Klebsiella pneumoniae* is an important zoonotic opportunistic pathogen. Of particular concern is the emergence of multidrug‐resistant hypervirulent *K. pneumoniae* (MDR‐hvKP), which represents a serious public health threat. Bacteriophage (phage) therapy has emerged as a promising solution to combat antibiotic‐resistant *K. pneumoniae* infections. Here, we characterize a novel lytic phage, vB_Kp_H122, that specifically targets K57 capsular‐type MDR‐hvKP. vB_Kp_H122 exhibited the characteristic morphology of a siphovirus and demonstrated efficient infection kinetics, with an optimal multiplicity of infection (MOI) of 0.001 and a latent period of ~5 min. It also demonstrated considerable stability across a pH range of 4–11 and at temperatures from 4°C to 50°C, as well as potent activity against *K. pneumoniae* biofilms. The phage has a linear double‐stranded DNA genome of 46,077 bp with a G + C content of 47.61% and belongs to a novel species within the genus *Roufvirus*. Its genome contained no identifiable genes associated with lysogeny, virulence, or antibiotic resistance, supporting its therapeutic safety. In a mouse infection model, a single dose of vB_Kp_H122 at 2 × 10^6^ PFU significantly reduced bacterial loads in organs, alleviated pathological damage, and provided complete protection against lethal challenge with K57 MDR‐hvKP. These findings suggest that vB_Kp_H122 may have potential as an antibacterial candidate against K57 MDR‐hvKP isolates.

## 1. Introduction

*Klebsiella pneumoniae*, a Gram‐negative pathogen within the *Enterobacteriaceae* family, is a natural inhabitant of the gastrointestinal microbiome of healthy humans and animals [1]. However, this important opportunistic pathogen also causes severe infectious diseases, including urinary tract infections, surgical site infections, pneumonia, and septicemia, in immunocompromised individuals. Furthermore, the emergence of hypervirulent *K. pneumoniae* (hvKP) isolates has led to community‐acquired invasive syndromes, causing life‐threatening conditions, such as pyogenic liver abscess complicated by meningitis [2]. HvKP is a distinct pathotype characterized by enhanced invasive virulence, driven by specific virulence plasmids and the presence of key biomarkers, including *iucA*, *iroB*, *peg-344*, *rmpA*, and *rmpA2* [3, 4]. Most hvKP strains exhibited the hypermucoviscous phenotype, which is typically associated with increased capsule production [5]. While historical serotyping defined at least 79 capsule types in *K. pneumoniae*, current capsular typing relies on molecular methods such as PCR‐based genotyping [6]. Among them, K1, K2, K5, K16, K20, K54, and K57 are most commonly associated with hvKP [7]. The prevalence of hvKP varies considerably; in endemic regions, such as China, it has been reported to reach as high as 12%–45% [8].

*K. pneumoniae* also ranks among the most significant bacterial pathogens due to its ability to resist antibiotics and is classified as one of the ESKAPE pathogens [1, 9]. The emergence of carbapenem‐resistant and extended‐spectrum *β*‐lactamase (ESBL)‐producing *K. pneumoniae* strains is a major global concern. Initially, hvKP and multidrug‐resistant *K. pneumoniae* emerged as distinct evolutionary pathotypes [10]. However, the boundaries between these two pathotypes are increasingly blurring [11]. In recent years, multidrug‐resistant hypervirulent *K. pneumoniae* (MDR‐hvKP), a new superbug combining hypervirulence with multidrug resistance, has emerged and is now spreading globally [11]. K57, one of the well‐known hvKP capsular types, is closely associated with pyogenic liver abscess and invasive infections in humans and animals [7]. *K. pneumoniae* K57 is also a common capsular type in dairy cows with mastitis in China. According to two independent surveys covering 13 and 14 provinces of China, respectively, K57 was identified as the most prevalent capsular type, with detection rates of 45% in both studies [12, 13]. Moreover, the K57 strains are exhibiting evolutionary convergence of multidrug resistance and hypervirulence, showing a trend toward increased resistance to most commonly used antibiotics compared with K1 and K2 capsular types [14]. Among *K. pneumoniae* strains collected from the Moscow Neurosurgery ICU (2014–2019), K57 accounted for 18.6% of isolates, ranking as the second most prevalent capsular type [15]. Notably, more than half of the hvKP isolates were hybrid MDR‐hvKP strains, with one K57 strain carrying not only *bla*_CTX-M-55_ but also two carbapenemase genes, *bla*_OXA-48_ and *bla*_NDM-1_ [15]. Another study documented that a K57 hvKP strain, already harboring a pLVPK‐like virulence plasmid, evolved into MDR‐hvKP through the acquisition of a conjugative *bla*_KPC-2_‐encoding plasmid [16]. The ongoing emergence of such “superbugs”, which are armed with both antimicrobial resistance and enhanced virulence, poses a serious threat and necessitates the development of innovative approaches to combat them.

Bacteriophages, also known as phages, are viruses that lyse and kill bacterial hosts. As a promising therapeutic alternative, bacteriophages possess distinct advantages, including host specificity and self‐amplification capability at infection sites, and have demonstrated minimal side effects in therapies to date [17]. Phage therapy is attracting growing attention for its demonstrated potential in the treatment of *K. pneumoniae* infections [18]. However, most of the identified *K. pneumoniae* bacteriophages are highly specific and only exhibit lytic activity against particular capsular types [19]. This narrow spectrum of activity makes the selection of appropriate phage therapy a significant challenge against the extensive capsule diversity of *K. pneumoniae*. Therefore, expanding the *K. pneumoniae* phage resource library with a wide array of characterized bacteriophages is essential. Although numerous *K. pneumoniae* phages targeting the prevalent capsular types K1 and K2 have been isolated, phages active against K57 strains remain limited, with only a few studies reported to date [20].

In this study, a lytic bacteriophage, designated vB_Kp_H122, was isolated using a multidrug‐resistant clinical isolate of *K. pneumoniae* K57 capsular type. Following its isolation, phage vB_Kp_H122 was characterized for its biological properties and genomic features through whole‐genome sequencing and bioinformatic analysis. The in vivo bactericidal efficacy of vB_Kp_H122 was further evaluated in a mouse model, establishing it as a promising therapeutic candidate against K57 MDR‐hvKP.

## 2. Material and Methods

### 2.1. Bacterial Strains and Growth Conditions

All *K. pneumoniae* strains used in this study and their sources are listed in Table 1. *K. pneumoniae* was routinely cultured in Luria–Bertani (LB) broth (Difco). The Kaptive 2.0 tool was used for capsular typing of all 34 *K. pneumoniae* strains [21]. Virulence genes of host strain M12 were analyzed using the Virulence Factor Database (http://www.mgc.ac.cn/VFs/). Identification of hvKP was based on a combined assessment of the hypermucoviscous phenotype (string test ≥5 mm) and the presence of key biomarkers, including *iucA*, *iroB*, *peg-344*, *rmpA*, and *rmpA2*. Strains exhibiting both multidrug resistance and hvKP characteristics were classified as MDR‐hvKP. Multilocus sequence typing of host strain M12 was conducted with Kleborate (https://github.com/katholt/Kleborate) based on the seven housekeeping genes (*gapA*, *infB*, *mdh*, *pgi*, *phoE*, *rpoB*, and *tonB*). The agar disk diffusion method was used to determine the antimicrobial susceptibility of the *K. pneumoniae* M12 strain to cefazolin (30 µg), ceftriaxone (30 µg), ceftazidime (30 µg), imipenem (10 µg), meropenem (10 µg), doxycycline (30 µg), ciprofloxacin (5 µg), streptomycin (10 µg), and fosfomycin (200 µg). The interpretation of results followed the Clinical and Laboratory Standards Institute Performance Standards for Antimicrobial Susceptibility Testing document M100‐S33 [22]. For polymyxin B, susceptibility testing was performed using the broth dilution method, with results interpreted according to the European Committee on Antimicrobial Susceptibility Testing (EUCAST) breakpoints (http://www.eucast.org/clinical_breakpoints/).

**Table 1:** The host range of bacteriophage vB_Kp_H122.

Strain	Capsule locus	Source	Plaque assay
NTUH K2044	KL1	Human	−
ATCC 43816	KL2	Human	−
ATCC 13883	KL3	Human	−
H51	KL5	Cow	−
M198‐3	KL10	Human	−
H52	KL13	Cow	−
T22	KL14	Human	−
KP2339	KL15	Pig	−
CT01	KL16	Pig	−
NPM	KL25	Pig	−
P114	KL27	Human	−
M146	KL30	Human	−
CC1	KL31	Pig	−
P1762	KL35	Human	−
KP117	KL36	Pig	−
Lan1	KL54	Pig	−
L4	KL56	Cow	−
M139	KL57	Human	+
M110	KL57	Human	+
SS139	KL57	Human	+
P139	KL57	Human	+
CKP1	KL57	Chicken	+
CKP2	KL57	Chicken	+
CKP4	KL57	Chicken	+
CKP6	KL57	Chicken	+
CKP10	KL57	Chicken	+
M51	KL57	Cow	+
M12	KL57	Cow	+
CC12	KL57	Cow	+
CKP3	KL63	Chicken	−
KP11	KL64	Human	−
KP7	KL81	Pig	−
M187	KL102	Human	−
T18	KL122	Human	−

*Note:* “+” signifies the formation of transparent plaques; “−” indicates no plaque formation.

### 2.2. Experimental Animals

Female SPF‐grade BALB/c mice aged 6 weeks were obtained from Beijing Vital River Laboratory Animal Technology Co., Ltd. (Beijing, China). The mice were housed in individually ventilated cages under standard conditions with free access to food and water. The procedure for the animal experiments in this study was approved by the Animal Ethics Committee of the Harbin Veterinary Research Institute of the Chinese Academy of Agricultural Sciences (approval number HVRI‐IACUC‐24121102GR).

### 2.3. Phage Isolation and Purification

Phages were isolated using the *K. pneumoniae* M12 strain as the host strain, following the method reported by Wang et al. [23] with some modifications. Briefly, samples collected from farm sewage were centrifuged at 8000 × *g* for 20 min at 4°C, and the supernatants were filtered through a 0.22‐µm‐pore‐size membrane (Millipore, USA). MDR‐hvKP M12 was cultured in LB medium to an optical density at 600 nm (OD_600_) of 0.6. The filtrate sample (50 mL) was mixed with bacterial culture (10 mL) of MDR‐hvKP M12 and coincubated with 2× concentrated LB (50 mL) for 12 h at 37°C. The culture was centrifuged (12,000×*g*, 5 min), and the supernatant was filtered through a 0.22‐µm‐pore‐size membrane. Phage activity was determined using the double‐layer agar method. A single plaque was picked from the plate using a sterile pipette and soaked in sodium chloride‐magnesium sulfate (SM) buffer (100 mM NaCl, 50 mM Tris, 10 mM MgSO_4_, and 0.01% gelatin, pH 7.5). This process was repeated at least five times to obtain the purified phage. The final phage stock, with a titer of 2 × 10^10^ PFU/mL, was stored at 4°C.

### 2.4. Transmission Electron Microscopy (TEM)

The phage suspension (10^9^ PFU/mL) was placed on carbon‐coated copper grids. After incubation for 10 min, the phage particles were negatively stained with 2% phosphotungstic acid for 20 s. The phage morphology was observed using a Hitachi H‐7650 transmission electron microscope (Hitachi, Tokyo, Japan).

### 2.5. Host Range Determination

The host range of phage vB_Kp_H122 was defined via the spot test assay as previously described [24], using a panel of 34 *K. pneumoniae* strains (Table 1). Briefly, an exponential‐phase bacterial culture adjusted to 10^8^ CFU/mL was added to 0.6% LB agar and overlaid onto 1.5% LB agar plates. Subsequently, 5 µL of phage suspension was spotted onto the upper agar layer, and plates were incubated at 37°C for 12 h. Strains producing clear, distinct plaques at the inoculation site were defined as susceptible to the phage. The experiments were performed in triplicate.

### 2.6. Determination of the Optimal MOI

The multiplicity of infection (MOI) is defined as the ratio of phage particles to host bacterial cells at the initial infection stage (MOI = plaque‐forming units [PFU]/colony‐forming units [CFU]) [25]. For determination of the optimal infection for the phage and host bacterium, M12 was grown to the logarithmic phase (optical density at 600 nm = 0.6) and adjusted to ~10^8^ CFU/mL. The phage was mixed with the bacterial culture at MOI values of 0.0001, 0.001, 0.01, 0.1, 1, 10, and 100. The mixtures were supplemented with 10 mL of LB broth, incubated at 37°C for 4 h, and centrifuged at 12,000 × *g* for 10 min at 4°C. The supernatants containing phage particles were collected, and phage titers were determined by the double‐layer agar method. The MOI yielding the highest progeny phage titer was identified as the optimal MOI.

### 2.7. One‐Step Growth Assay

The one‐step growth curve was determined as previously described [26]. Briefly, the midlog growth phase culture of the MDR‐hvKP M12 strain was adjusted to 10^8^ CFU/mL and mixed with phage solution at an MOI of 0.001. The phage was allowed to adsorb for 10 min at 37°C and then washed with LB medium via centrifugation at 12,000 × *g* for 5 min to remove the unabsorbed phage. The precipitate was resuspended in 20 mL LB broth and incubated at 37°C with shaking. Samples were collected at 5 min intervals for 60 min, and phage titers were quantified by the double‐layer agar method. The experiments were performed in triplicate. The latent period was defined as the interval from infection to the first release of progeny phages. The burst period was defined as the interval during which the phage titer increased rapidly before reaching the plateau period. Burst size is calculated as the total number of phage progeny released divided by the number of infected cells [27].

### 2.8. Phage Adsorption Assay

To examine the adsorption rate of phage to host bacterium, the phage and MDR‐hvKP M12 were mixed at the optimal MOI and incubated at 37°C. At various time points throughout the 30‐minute period, 100 μL samples were taken from the phage–bacteria mixture and immediately centrifuged at 12,000×*g* for 5 min at 4°C. The supernatant containing unabsorbed phages was diluted and quantified using the double‐layer agar plate method. The adsorption curve was constructed using the ratio of adsorbed phages at different time intervals to the number of initial phages. The experiments were performed in triplicate.

### 2.9. Time‐Kill Assay

The bactericidal efficiency of the phage was evaluated in vitro against its host *K. pneumoniae* M12, as previously described [28]. Phages were inoculated into bacterial culture (1 × 10^8^ CFU/mL) at MOIs of 0, 0.001, 0.01, 0.1, and 1, followed by 7 h of incubation at 37°C. During this period, bacterial growth kinetics were monitored by measuring the optical density at 600 nm at hourly intervals. The experiments were performed in triplicate.

### 2.10. Thermal and pH Stability Assays

To investigate the thermal stability of the phage, 500 µL of the phage suspension (1 × 10^9^ PFU/mL) was incubated for 2 h at 4, 25, 37, 50, 60, 70, and 80°C. For the pH stability assay, the phage solutions were subjected to incubation at 37°C for 2 h in SM buffer with various pH values ranging from 2.0 to 13.0. Phage titers following incubation under different temperature and pH conditions were determined by the double‐layer agar method. The experiments were performed in triplicate.

### 2.11. Evaluation of Phage Antibiofilm Activity

The effectiveness of phage vB_Kp_H122 against *K. pneumoniae* biofilms was evaluated using the crystal violet assay. Briefly, the MDR‐hvKP isolate M12, at a final concentration of 1 × 10^6^ CFU/mL, was diluted in Tryptic Soy Broth containing phage at MOIs ranging from 0.01 to 1 in the wells of a 24‐well polypropylene microtiter plate. The plate was statically incubated at 37°C for 24 h. For the biofilm removal assay, preformed biofilms were incubated with phages at MOIs ranging from 0.01 to 1 for 12 h at 37°C. The wells were washed twice with PBS, air‐dried, and stained with 0.1% crystal violet solution for 30 min. After staining, a 33% (v/v) glacial acetic acid solution was added to each well to solubilize the stained biofilms, and the absorbance values at a wavelength of 570 nm were measured using an EnVision Multimode Plate Reader (PerkinElmer, Waltham, USA). The quantification of viable cells in biofilm was performed using plate colony counts. The experiments were performed in triplicate.

### 2.12. Phage Genome Sequencing and Bioinformatic Analysis

Phage genomic DNA was extracted using the viral DNA extraction kit (Tiangen Biotech, Beijing, China) according to the manufacturer’s protocol. Whole‐genome sequencing was performed by Personalbio Technology Co., Ltd. (Shanghai, China) using an Illumina NovaSeq sequencing platform. *De novo* genome assembly was conducted using the SPAdes 3.12.0 version software [29]. Open reading frames (ORFs) were predicted using the RAST server (http://rast.nmpdr.org/) and validated with GeneMark [30, 31]. Homology of the predicted phage proteins was determined through BLASTp analysis [32], while genome similarity was assessed via BLASTn [33]. Virulence factors were identified through the Virulence Factor Database (VFDB, http://www.mgc.ac.cn/VFs/), and antibiotic resistance genes were screened against the Comprehensive Antibiotic Resistance Database (CARD, https://card.mcmaster.ca/) [34, 35]. The circular genome map was generated using CGView (https://proksee.ca/) to visualize genomic features [36]. For taxonomic classification, a neighbor‐joining phylogenetic tree was constructed with MEGA 11, comparing vB_Kp_H122 with published phage genomes [37]. Bootstrap analysis was used to evaluate the reliability of the phylogenetic tree, and a total of 1000 bootstrap replicates were performed. Comparative genomic analysis with closely related phages (selected based on BLASTn identity and coverage) was performed using Easyfig to reveal syntenic relationships [38].

### 2.13. Phage Therapy in the Mouse Bacteremia Model

The therapeutic efficacy of the phage against *K. pneumoniae* was evaluated in vivo using a mouse bacteremia model, as previously described [39, 40]. Forty 6‐week‐old female BALB/c mice were randomly assigned to four groups (*n* = 10 per group) using a random number table. No blinding was performed in this animal experiment. All groups received an intraperitoneal injection of 2 × 10^7^ CFU of MDR‐hvKP strain M12 to establish infection models. One h after the bacterial challenge, the phage treatment groups received single intraperitoneal doses of phages at concentrations of 2 × 10^5^, 2 × 10^6^, and 2 × 10^7^ PFU, respectively. The negative control group received an equal volume of PBS buffer. Mouse mortality following phage treatment was monitored for 7 days.

### 2.14. Assessment of Bacterial Load and Histopathological Changes

To monitor the bacterial load in vivo, 24 6‐week‐old female BALB/c mice were randomly divided into two groups (*n* = 12 per group) using a random number table: a phage‐treated group and a control group. No blinding was performed in this animal experiment. All mice were intraperitoneally infected with 5 × 10^6^ CFU of *K. pneumoniae* M12. At 1 h postinfection, mice in the phage‐treated group received 100 μL of phage suspension containing 2 × 10^6^ PFU, whereas the control group received an equal volume of PBS buffer. At 24 and 48 h post‐treatment, the liver, spleen, lungs, and kidneys from six mice per group were aseptically harvested and homogenized. The homogenates were subsequently subjected to serial dilution and counted using LB agar. For histopathological examination, tissue samples from the collected organs were immediately fixed in 4% paraformaldehyde. After routine dehydration, paraffin embedding and sectioning, all tissue sections were stained with hematoxylin and eosin (H&E). The pathological lesions of each organ were observed and photographed under an optical microscope.

### 2.15. Statistical Analysis

Statistical analyses were performed using GraphPad Prism 8.0 (GraphPad Software, San Diego, CA, USA). Results are expressed as mean ± standard deviation (SD). Statistical significance was determined via unpaired *t*‐tests or one‐way analysis of variance (ANOVA). A *p*‐value < 0.05 was considered statistically significant.

## 3. Results

### 3.1. Phage Isolation and Morphology

**Figure 1:**
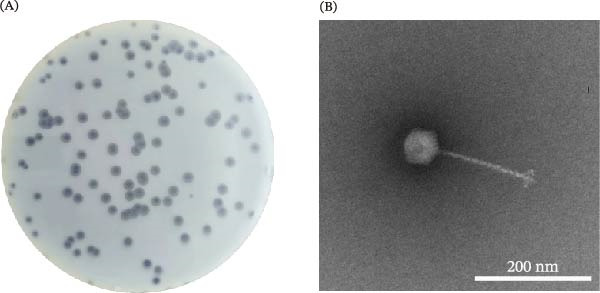
Morphological characterization of phage vB_Kp_H122. (A) Plaque morphology of phage vB_Kp_H122 on the lawn of *K. pneumoniae* strain M12. (B) Transmission electron micrograph of bacteriophage vB_Kp_H122. The scale bar is 200 nm.

The *K. pneumoniae* M12 was identified as capsular type K57 and MLST type ST412. It exhibited a hypermucoviscous phenotype (string ≥ 5 mm) on the blood agar plate. This strain was found to harbor key virulence genes, including *rmpA*, *rmpA2*, *iucA*, *iroB*, and *peg344*. Antimicrobial susceptibility testing showed that the corresponding inhibition zone diameters (mm) were 13 for cefazolin (30 µg), 23 for ceftriaxone (30 µg), 24 for ceftazidime (30 µg), 28 for imipenem (10 µg), 29 for meropenem (10 µg), 9 for doxycycline (30 µg), 18 for ciprofloxacin (5 µg), 20 for streptomycin (10 µg), and 8 for fosfomycin (200 µg). The MIC value of polymyxin B was 8 μg/mL. According to CLSI and EUCAST criteria, strain M12 exhibited resistance to cefazolin, doxycycline, ciprofloxacin, fosfomycin, and polymyxin, indicating that it qualifies as an MDR‐hvKP isolate. Using a K57‐capsular‐type MDR‐hvKP strain M12, a novel lytic bacteriophage named vB_Kp_H122 was isolated and purified from wastewater. This phage exhibits strong lytic activity against the host strain M12, forming large plaques with a central clear zone and surrounding halos (Figure 1A), a morphological trait indicative of a phage capable of hydrolyzing the capsule of the host bacterium. Following the preparation of high‐titer phage stocks, TEM revealed that the phage vB_Kp_H122 possessed an isometric polyhedral capsid (60 nm in diameter) and a noncontractile tail (170 nm in length) (Figure 1B), which is a typical morphological characteristic of siphoviruses.

### 3.2. Host Range Determination

The host range of phage vB_Kp_H122 was determined against a panel of 34 *K. pneumoniae* isolates obtained from various sources using a plaque assay. As shown in Table 1, phage vB_Kp_H122 could lyse 12 of the 34 tested strains, producing clear plaques. All 12 susceptible strains belonged to the K57 capsular type, while no lytic activity was observed against the *K. pneumoniae* strains of other capsular types (Table 1). These results demonstrate that phage vB_Kp_H122 exhibits highly specific lytic activity against *K. pneumoniae* K57 capsular type.

### 3.3. Biological Characteristics of Phage vB_Kp_H122

**Figure 2:**
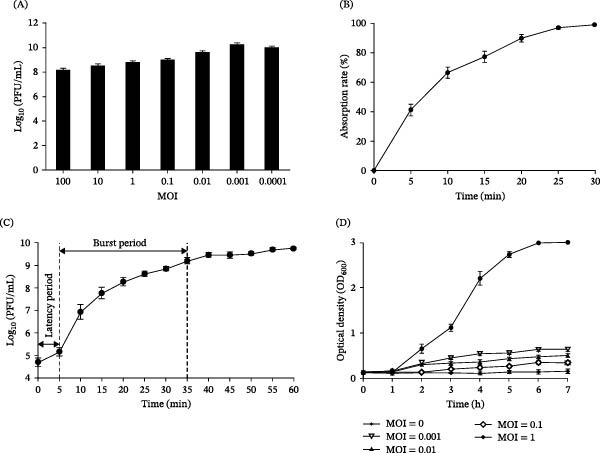
Biological characteristics of phage vB_Kp_H122. (A) Optimal multiplicity of infection determination for phage vB_Kp_H122. Burst size is calculated as the total number of phage progeny released divided by the number of infected cells. (B) Adsorption curve of phage vB_Kp_H122. (C) One‐step growth curve of phage vB_Kp_H122. (D) Bactericidal curve of phage vB_Kp_H122. Data are shown as the mean ± SD from three independent replicates.

The optimal MOI for phage vB_Kp_H122 was determined by coincubating it with its host, MDR‐hvKP M12, across a range of MOI values. The highest titer of progeny phages was observed at an MOI of 0.001 (Figure 2A), identifying this as the optimal MOI for maximizing progeny release during vB_Kp_H122 replication. The adsorption kinetics of phage vB_Kp_H122 to its host were characterized by its adsorption curve, with over 60% of phages adsorbed within 10 min and 90% of phages adsorbed within 20 min (Figure 2B). Subsequently, a one‐step growth curve was performed to characterize the infection cycle of phage vB_Kp_H122 in its host strain M12. The result showed that vB_Kp_H122 has a short latency period of 5 min and a burst size of 154 PFU per infected cell after the plateau period (Figure 2C), indicating its efficient replication capacity. The bactericidal efficacy of phage vB_Kp_H122 against MDR‐hvKP M12 was assessed in vitro at different MOI values. As shown in Figure 2D, the uninfected control culture displayed normal growth, with OD_600_ increasing logarithmically and progressing to the stationary phase by 7 h. However, phage addition at MOIs of 0.001, 0.01, 0.1, and 1 caused a marked reduction in bacterial density, where the lytic effect strengthened with increasing the MOI. These results showed that phage vB_Kp_H122 exhibits strong bactericidal activity against MDR‐hvKP strain M12.

### 3.4. Thermal and pH Stability of Phage vB_Kp_H122

**Figure 3:**
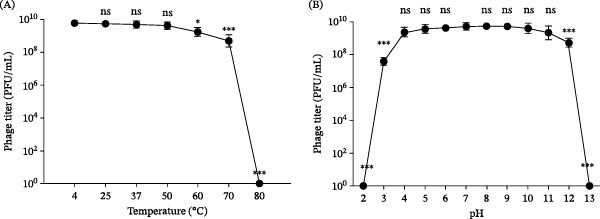
Stability of phage vB_Kp_H122 under varying thermal and pH conditions. (A) Thermal stability: phage titers were determined after incubation at 4–80°C for 2 h. (B) pH stability: phage titers were determined after incubation at pH (2.0–13.0) at 37°C for 2 h. Data represent the mean ± SD from three independent replicates. Statistical significance was calculated via one‐way ANOVA, *p* > 0.05 (ns), *p* < 0.05 ( ^∗^), *p* < 0.001 ( ^∗∗∗^).

To evaluate its thermal stability, phage vB_Kp_H122 was incubated at various temperatures for 2 h, and the phage titer was measured. As shown in Figure 3A, the phage titer of vB_Kp_H122 remained virtually unchanged across a broad temperature range (4–50°C) but progressively declined at 60°C and was completely inactivated at 80°C. The stability of phage vB_Kp_H122 was further examined under various pH conditions (Figure 3B). The phage remained stable over a broad range (pH 4–11), with phage titers exhibiting minimal reduction (≤0.5 log_10_). Partial inactivation was observed at pH 3 and 12, while complete inactivation occurred under extreme conditions of pH 2 and 13. These findings demonstrate that phage vB_Kp_H122 exhibits robust stability across temperatures ranging from 4 to 50°C and a broad pH spectrum of 4.0–11.0.

### 3.5. Antibiofilm Activity of Phage vB_Kp_H122

**Figure 4:**
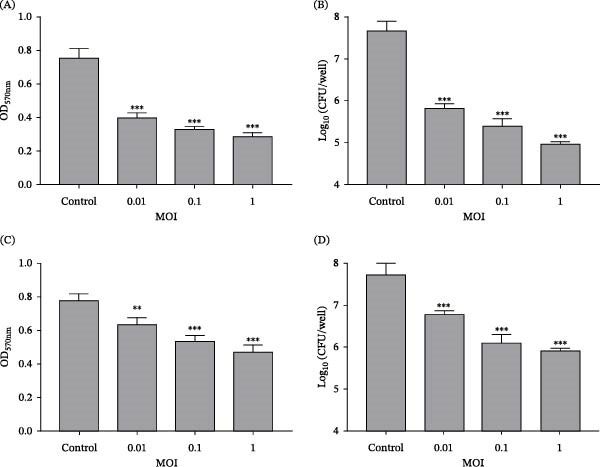
Antibiofilm effects of phage vB_Kp_H122 against *K. pneumoniae*. (A, B) Biofilm inhibition by vB_Kp_H122. The MDR‐hvKP isolate M12 was treated with vB_Kp_H122 at MOIs ranging from 0.01–1, and the mixtures were incubated at 37°C for 24 h. (C, D) Biofilm removal by vB_Kp_H122. Preformed mature biofilms were exposed to vB_Kp_H122 at MOIs ranging from 0.01 to 1 for 12 h. The antibiofilm activity of vB_Kp_H122 against M12 isolate was assessed using (A, C) crystal violet staining and (B, D) viable cell counting. Data represent the mean ± SD from three independent replicates. Statistical significance was calculated via one‐way ANOVA, *p*  < 0.01 ( ^∗∗^), *p* < 0.001 ( ^∗∗∗^).

To explore the antibiofilm efficacy of phage vB_Kp_H122, the MDR‐hvKP strain M12 was treated with varying concentrations of the phage, and biofilm biomass was assessed using crystal violet staining. After 24 h of incubation, phage treatment at MOIs of 0.01, 0.1, and 1 resulted in dose‐dependent reductions in biofilm biomass of ~67.1%, 79.9%, and 87.9%, respectively, compared to the untreated control (Figure 4A). In addition, biofilm cell viability was assessed through the quantification of CFUs on separated plates. Treatment with the phage at MOIs of 0.01, 0.1, and 1 reduced the viable bacterial count in the biofilm by 1.85, 2.27, and 2.71 log_10_, respectively, relative to the untreated control (Figure 4B). Furthermore, treatment of mature biofilms with phages at MOIs of 0.01, 0.1, and 1 for 12 h resulted in biofilm removal rates of 25.2%, 42.7%, and 54.5%, respectively, accompanied by a reduction in viable bacterial counts within the biofilms by 0.95, 1.63, and 1.81 log_10_ CFU, respectively (Figure 4C,D). The results indicate that phage vB_Kp_H122 can effectively inhibit biofilm formation and eradicate preformed biofilm of MDR‐hvKP M12.

### 3.6. Genome Analysis of Phage vB_Kp_H122

**Figure 5:**
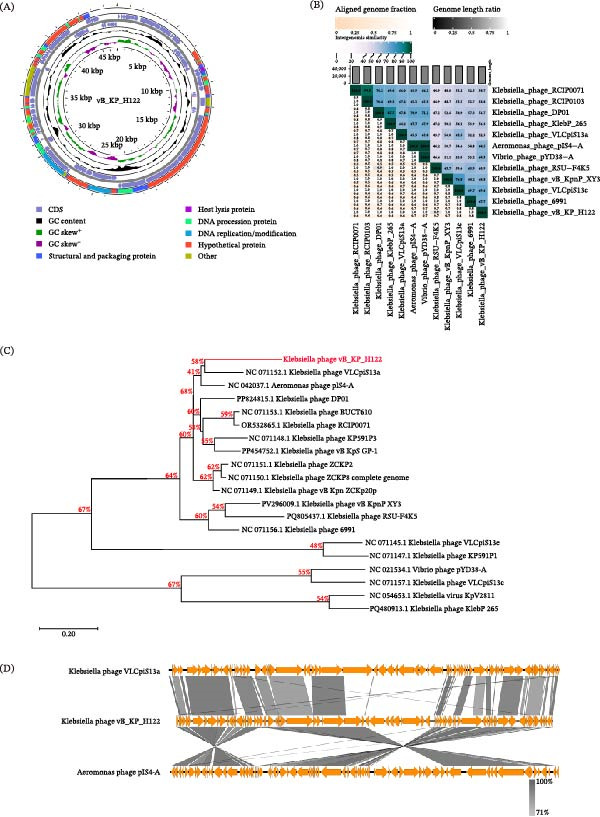
Genomic analysis of phage vB_Kp_H122. (A) Circular genome map of phage vB_Kp_H122. The annotated protein functions are categorized by their roles, with distinct colors assigned to each functional module. (B) Intergenomic similarity between the phage vB_Kp_H122 and the most similar phages was calculated using VIRIDIC. (C) Phylogenetic tree of phage vB_Kp_H122. The diagram was constructed with the complete phage genome sequences using the neighbor‐joining method in MEGA 11. The percentage values at tree nodes represent bootstrap support values. (D) Genome linear comparison of phages vB_Kp_H122, VLCpiS13a, and pIS4‐A.

The genome of phage vB_Kp_H122 was assembled into a single complete contig using SPAdes software (version 3.12.0), which is a linear double‐stranded DNA of 46,077 bp with a G + C content of 47.61% (Figure 5A). Annotation of the vB_Kp_H122 genome using bioinformatic software revealed a total of 79 ORFs. The ORFs accounted for a total of 42,837 bp, with a gene density as high as 92.97%. Among these ORFs, 29 ORFs were predicted to code for functional proteins, and 50 ORFs were predicted to code for hypothetical proteins. All the predicted ORFs were associated with phage structural, DNA packaging, host lysis, nucleic acid replication and metabolic regulation, and hypothetical proteins (Figure 5A). Notably, no antibiotic resistance genes, virulence genes, tRNA genes, or genes involved in lysogenic cycles were detected within the vB_Kp_H122 genome. VIRIDIC analysis clustered vB_Kp_H122 with phages belonging to the *Roufvirus* genus, with intergenomic similarities ranging from 52.5% to 69.6% in the genomes of the 11 closely related phages (Figure 5B). Phylogenetic analysis revealed that vB_Kp_H122 formed a distinct phylogenetic branch but showed close relatedness to the *K. pneumoniae* phage VLCpiS13a (accession number: NC 071152.1) and the *Aeromonas* phage pIS4‐A (accession number: NC_042037.1), with intergenomic similarities of 52.5% and 64.5%, respectively (Figure 5B,C). As shown by linear genome comparison, vB_Kp_H122, VLCpiS13a, and pIS4‐A exhibited substantial differences in their genomic arrangements despite sharing similarities in genome structure and functional genes (Figure 5D). With a whole‐genome nucleotide identity below the 95% species demarcation threshold established by the ICTV, bacteriophage vB_Kp_H122 is classified as a novel species within the genus *Roufvirus*, class *Caudoviricetes*.

### 3.7. In Vivo Therapeutic Evaluation of Phage vB_Kp_H122 in a Mouse Infection Model

**Figure 6:**
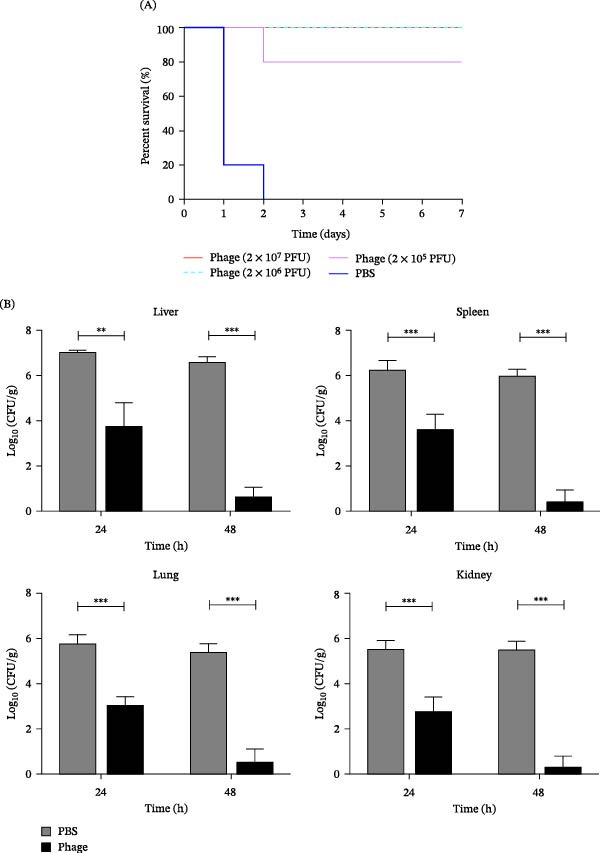
Phage vB_Kp_H122 rescued mice from K57‐type MDR‐hvKP infection. (A) Survival curves of mice challenged with MDR‐hvKP M12 (2 × 10^7^ CFU) and subsequently treated with varying doses of phage vB_Kp_H122, as shown in the figure. (B) Bacterial loads in liver, spleen, lung, and kidney from mice infected with MDR‐hvKP strain M12 (5 × 10^6^ CFU) and treated with phage vB_Kp_H122 (2 × 10^6^ PFU). Data are shown as the mean ± SD from six mice. Statistical significance was calculated via unpaired *t* tests, *p*  < 0.01 ( ^∗∗^), *p* < 0.001 ( ^∗∗∗^).

The efficacy of the phage in treating *K. pneumoniae* infection was assessed using a mouse bacteremia model. A dose of 2 × 10^7^ CFU per mouse of *K. pneumoniae* M12, administered intraperitoneally, was sufficient to induce 100% mortality within 48 h post infection. Mice infected with *K. pneumoniae* for 1 h were treated with various concentrations of 2 × 10^5^, 2 × 10^6^, and 2 × 10^7^ PFU of vB_Kp_H122. As shown in Figure 6A, all mice in the control group treated with PBS died within 48 h postinfection. In contrast, phage treatment conferred dose‐dependent protection; whereas the lower dose (2 × 10^5^ PFU) yielded an 80% survival rate, the two higher doses (2 × 10^6^ and 2 × 10^7^ PFU) both achieved 100% survival rates.

For organ bacterial load assessment, a dose of 5 × 10^6^ CFU was administered to mice because it enabled stable bacterial colonization in target organs without early mortality, thereby allowing phage‐mediated clearance to be assessed at the designated time points (24 and 48 h post‐treatment). At each endpoint, viable bacterial loads in tissue samples were quantified by CFU counts. Significant reductions in bacterial loads were observed in the liver, spleen, lung, and kidney following vB_Kp_H122 treatment, with reductions of 3.25, 2.62, 2.71, and 2.75 log_10_ CFU at 24 h, which further increased to 5.95, 5.54, 4.83, and 5.17 log_10_ CFU by 48 h, respectively (Figure 6B).

**Figure 7:**
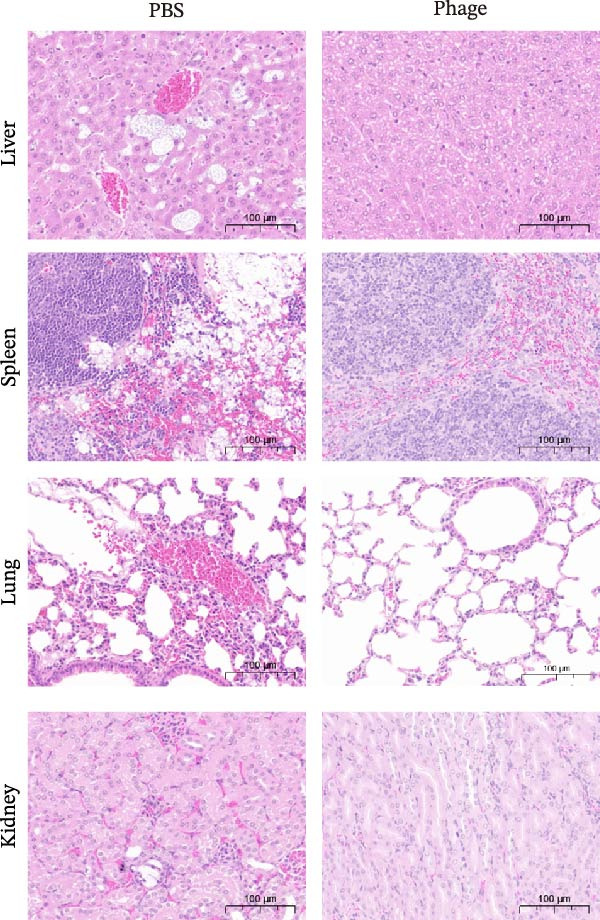
Histopathological evaluation of organ injury in infected mice treated with phage vB_Kp_H122. Tissue samples of the liver, spleen, lung, and kidney were collected from mice following PBS or vB_Kp_H122 treatment, fixed in 4% formalin, and subsequently sectioned for H&E staining. The scale bar is 100 μm.

Comparative histopathological examination was employed to evaluate the differences in the severity of tissue lesions between mice in the vB_Kp_H122‐treated and PBS control groups. As shown in Figure 7, H&E staining demonstrated severe pathological damage in PBS‐treated mice infected with *K. pneumoniae*. The predominant pathological changes included hepatic vacuolar degeneration with hepatocellular necrosis, extensive splenic vacuolar fusion accompanied by red/white pulp disruption and hemorrhage, pulmonary perivenular edema with mild inflammatory infiltration, and multifocal irregular hemorrhages in the renal medulla. In contrast to the PBS controls, vB_Kp_H122 treatment markedly attenuated pathological damage, with no evident injury observed in the four examined organs. These findings indicate that phage vB_Kp_H122 treatment attenuates *K. pneumoniae*‐induced tissue damage in this mouse model.

## 4. Discussion

The evolution of *K. pneumoniae* towards increased virulence and antimicrobial resistance represents an alarming trend, positioning it among the most pressing challenges in the control of Gram‐negative bacterial pathogens [41]. K57, a well‐known hvKP capsular type that is prevalent in Chinese dairy cows with mastitis and linked to serious human infections [8, 12, 13], is now also evolving into MDR‐hvKP in clinical settings through the acquisition of multidrug resistance genes [15, 16]. The growing demand for novel antimicrobial agents has driven a renewed focus on the exploration of phage therapy. As natural bacterial predators, bacteriophages employ a bactericidal mechanism that is fundamentally different from antibiotics. The abilities of bacteriophages to kill multidrug‐resistant bacteria make them a promising therapeutic strategy against drug‐resistant bacterial infections [42, 43]. In this study, a lytic phage, vB_Kp_H122, was isolated from farm sewage. To evaluate its therapeutic potential, we conducted a comprehensive assessment of its biological properties, genomic analysis, in vitro antibacterial and anti‐biofilm activity, and in vivo therapeutic effect in a mouse infection model. Our findings indicate that phage vB_Kp_H122 is highly effective against K57 MDR‐hvKP.

A low MOI combined with a short incubation period offers a more cost‐effective strategy for large‐scale bacteriophage reproduction, thereby enhancing the production efficiency [44]. In this study, phage vB_Kp_H122 demonstrated an optimal MOI of 0.001, a latent period of 5 min, and a burst size of 154 PFU/cell. Compared with another K57‐targeting phage, vB_KpnP_ZX1 (30 min, 125 PFU/cell), vB_Kp_H122 demonstrated superior growth kinetics, characterized by a shorter latent period and a larger burst size [28]. Moreover, the kinetic parameters of phage vB_Kp_H122 surpass those of some non‐K57‐type *K. pneumoniae* phages, specifically vB_KlebPS_265 (15 min, 26 PFU/cell) and Kpph9 (11 min, 25 PFU/cell), indicating superior bactericidal efficacy [45, 46]. Dynamic antibacterial monitoring further demonstrated the potent bactericidal efficacy of phage vB_Kp_H122, as shown by effective bacterial suppression across a range of MOIs.

The therapeutic efficacy of phages is directly dependent on their environmental stability, particularly temperature and pH variations, making this a crucial determinant for their practical application [47]. In this study, phage vB_Kp_H122 demonstrated considerable stability across a broad temperature range (4–50°C). This robust thermotolerance aligns with the stability profiles of other *K. pneumoniae* phages, such as KpnP_ZX1 and Henu_2 [28, 48]. Furthermore, phage vB_Kp_H122 exhibited robust stability across a broad pH range of 4.0–11.0, a property that is advantageous for its practical application. This characteristic enhances phage resilience, thereby mitigating the risk of inactivation prior to therapeutic action [49, 50].

Genomic sequencing and analysis are essential for advancing phage therapeutics as they provide critical insights into phage biology while ensuring clinical safety. To this end, therapeutic candidates must be rigorously screened to exclude hazardous genetic elements, including lysogeny‐associated genes, antibiotic resistance genes, and virulence‐associated genes, that could potentiate infection risks [51]. Analysis of the vB_Kp_H122 genome demonstrated that the phage is free from genes coding for virulence, antibiotic resistance, and lysogenicity, establishing its potential as a safe therapeutic agent. Based on sequence homology analysis, ORF36 is predicted to encode a putative tail fiber protein/depolymerase. The presence of halo zones surrounding the plaques further suggests that this phage may possess capsule‐degrading activity. However, the precise function of ORF36 requires experimental verification in future studies.

In vivo antibacterial experiments demonstrated the lytic effectiveness of phage vB_Kp_H122 and its potential for combating infections caused by K57 MDR‐hvKP in a mouse bacteremia model. Lethal infection with K57‐type *K. pneumoniae* at 2 × 10^7^ CFU resulted in 100% mortality within 48 h in the control group. In contrast, mice receiving vB_Kp_H122 at doses of 2 × 10^6^ PFU or higher exhibited 100% survival over the observation period. Furthermore, administration of vB_Kp_H122 was associated with marked reductions in organ bacterial burdens and attenuated tissue pathological damage. However, a limitation of vB_Kp_H122 is its strict capsule‐dependent specificity. Host range testing confirmed that it only lyses K57‐type *K. pneumoniae*, with no activity against other clinically prevalent capsular types such as K1, K2, or K54. Given the high capsular diversity among clinical MDR‐hvKP isolates, our future studies will focus on further expanding the *K. pneumoniae* phage repository and exploring phage cocktail therapy. Another limitation of this study is that the resistance frequency of *K. pneumoniae* against vB_Kp_H122 was not determined. This parameter warrants systematic investigation in future studies to comprehensively evaluate the long‐term therapeutic viability of this phage.

## 5. Conclusion

In summary, this study reports a newly isolated lytic phage, vB_Kp_H122, which exhibits potent activity against K57 MDR‐hvKP. The phage demonstrates robust stability across temperatures ranging from 4°C to 50°C and a broad pH range of 4.0–11.0, a key characteristic that may facilitate its further application. In a mouse model, a single dose of phage vB_Kp_H122 provided complete protection against lethal K57 MDR‐hvKP challenge, significantly reducing bacterial loads and alleviating organ pathological damage. Collectively, these findings indicate that phage vB_Kp_H122 represents a valuable candidate for further evaluation against K57 MDR‐hvKP infections.

## Author Contributions

Fang Xie and Wanjiang Zhang conceived and designed the experiments. Cuilong Fan, Qiu Xu, Yaowei Liu, and Susu Du performed the experiments. Cuilong Fan, Susu Du, Xiaofeng Lu, Yuxuan Yuan, Longhua Lin, and Shuangyu Xie analyzed the data. Fang Xie, Cuilong Fan, Stefan Schwarz, and Wanjiang Zhang wrote the manuscript with input from all coauthors.

## Funding

This work was supported by the Prevention and Control of Emerging and Major Infectious Diseases‐National Science and Technology Major Project (Grant 2025ZD01900100) and the Central Public‐Interest Scientific Institution Basal Research Fund (Grant 1610302025002).

## Disclosure

All the authors have contributed to the manuscript and approved the submitted version.

## Ethics Statement

All animal experiments were reviewed and approved by the Animal Ethics Committee of the Harbin Veterinary Research Institute, Chinese Academy of Agricultural Sciences (approval number HVRI‐IACUC‐24121102GR). All procedures were in accordance with national laboratory animal welfare standards and institutional guidelines. Mice were anesthetized via intraperitoneal injection of sodium pentobarbital (100 mg/kg). For organ collection at the experimental endpoint, mice were humanely euthanized by cervical dislocation under anesthesia.

## Conflicts of Interest

The authors declare no conflicts of interest.

## Data Availability

The complete genome sequence of phage vB_Kp_H122 has been deposited in the figshare database (https://doi.org/10.6084/m9.figshare.29878109.v1).
